# Life History and Demographic Drivers of Reservoir Competence for Three Tick-Borne Zoonotic Pathogens

**DOI:** 10.1371/journal.pone.0107387

**Published:** 2014-09-18

**Authors:** Richard S. Ostfeld, Taal Levi, Anna E. Jolles, Lynn B. Martin, Parviez R. Hosseini, Felicia Keesing

**Affiliations:** 1 Cary Institute of Ecosystem Studies, Millbrook, New York, United States of America; 2 Department of Fisheries and Wildlife, Oregon State University, Corvallis, Oregon, United States of America; 3 Departments of Biomedical Sciences and Zoology, Oregon State University, Corvallis, Oregon, United States of America; 4 Department of Integrative Biology, University of South Florida, Tampa, Florida, United States of America; 5 EcoHealth Alliance, New York, New York, United States of America; 6 Biology Program, Bard College, Annandale-on-Hudson, New York, United States of America; Kansas State University, United States of America

## Abstract

Animal and plant species differ dramatically in their quality as hosts for multi-host pathogens, but the causes of this variation are poorly understood. A group of small mammals, including small rodents and shrews, are among the most competent natural reservoirs for three tick-borne zoonotic pathogens, *Borrelia burgdorferi, Babesia microti,* and *Anaplasma phagocytophilum*, in eastern North America. For a group of nine commonly-infected mammals spanning >2 orders of magnitude in body mass, we asked whether life history features or surrogates for (unknown) encounter rates with ticks, predicted reservoir competence for each pathogen. Life history features associated with a fast pace of life generally were positively correlated with reservoir competence. However, a model comparison approach revealed that host population density, as a proxy for encounter rates between hosts and pathogens, generally received more support than did life history features. The specific life history features and the importance of host population density differed somewhat between the different pathogens. We interpret these results as supporting two alternative but non-exclusive hypotheses for why ecologically widespread, synanthropic species are often the most competent reservoirs for multi-host pathogens. First, multi-host pathogens might adapt to those hosts they are most likely to experience, which are likely to be the most abundant and/or frequently bitten by tick vectors. Second, species with fast life histories might allocate less to certain immune defenses, which could increase their reservoir competence. Results suggest that of the host species that might potentially be exposed, those with comparatively high population densities, small bodies, and fast pace of life will often be keystone reservoirs that should be targeted for surveillance or management.

## Introduction

Blacklegged ticks (*Ixodes scapularis*) are the primary vectors responsible for transmitting a suite of zoonotic pathogens in eastern and central North America. Lyme disease, caused by the spirochete bacterium, *Borrelia burgdorferi*, is the most prevalent of these tick-borne diseases, with approximately 300,000 cases occurring annually in the United States [1]. Two other emerging tick-borne diseases are human babesiosis, caused by the piroplasm, *Babesia microti*, and human granulocytic anaplasmosis, caused by the rickettsial bacterium, *Anaplasma phagocytophilum*. Despite substantial phylogenetic separation, these three pathogens co-circulate in host and vector communities and show similar transmission dynamics [2], the causes of which we address here.

All three of these pathogens have a broad host range, routinely infecting most species in the community of terrestrial mammals and birds that have been tested [3]–[6]. For all three pathogens, ticks acquire infection when they take a blood meal from an infected host; transovarial transmission has not been demonstrated [7]–[10]. Therefore, considerable interest has focused on the capacity of different vertebrate host species to transmit infection to feeding ticks. The probability that a particular host species will transmit an infection to uninfected ticks under natural conditions has been termed “realized reservoir competence”. Realized reservoir competence is the product of the probability that a host is infected (prevalence) and the probability that it transmits given that it is infected (infectivity) [11]. When prevalence in a given host species is high, which is the case in many endemic zones, its realized reservoir competence and infectivity converge [11]. In regards to the above pathogens, realized reservoir competence is estimated by collecting fed larval blacklegged ticks after they have detached from a known host, allowing them to molt to the nymphal stage, and then testing nymphs individually for infection with each of the zoonotic agents (e.g., [3]). Any nymphs that test positive must have acquired the infection from the host from which they were collected.

For each of the three above pathogens, realized reservoir competence varies dramatically among vertebrate species [3]–[6]. But a striking pattern has emerged from recent studies in the hyperendemic zone for tick-borne zoonoses in the northeastern United States. Two rodents, the white-footed mouse (*Peromyscus leucopus*) and the eastern chipmunk (*Tamias striatus*), and two soricimorphs, the masked shrew (*Sorex cinereus*) and the northern short-tailed shrew (*Blarina brevicauda*), have among the highest realized reservoir competence values of all hosts for all three pathogens [3]–[6]. Here, we ask whether these hosts share attributes that facilitate the success of this group of very different zoonotic agents.

Life history theory suggests that species of animals and plants that invest heavily in growth and reproduction are subject to trade-offs that reduce allocation to self-maintenance, including immune defense [12]. Consequently, species with fast life histories (early maturity, rapid breeding, short longevity) might generally be more likely to become infected and be infectious than are their slower-lived counterparts, all else equal [13]–[20]. If investments in fast-paced lives indeed reduce allocation to immune defense, then relatively fast-lived species might be more competent reservoirs for multi-host pathogens [20]. Support for this expectation recently was found for the Lyme disease system by Previtali et al. [21], who examined some immune functions in white-footed mice, eastern chipmunks, and eastern gray squirrels (*Sciurus carolinensis*); these hosts show, respectively, high, moderate, and low reservoir competence for *B. burgdorferi*. Antibody responses to a novel antigen challenge were highest in squirrels, which have the slowest life history, and lowest in mice, which have the fastest life history [21]. Huang et al. [22] tested the hypothesis that the most competent reservoirs for the agent of Lyme disease are the species with the fastest life histories; for the nine host species they examined, reservoir competence was inversely related to body size. They found similar patterns for two mosquito-transmitted zoonotic pathogens. However, whether body size is somehow causally related to reservoir competence, or simply a correlate of other, more direct influences, is unknown.

In addition to the potential for host life history traits to influence reservoir competence via immune functioning, host traits might also influence the ability of pathogens to adapt to and exploit hosts. Because an extreme host generalist vectors them, all three of the tick-borne pathogens are distributed each generation to many species of hosts. Adaptation to evade immunity and proliferate in one host might impede the ability of the pathogen to infect and proliferate in other hosts [23]. Ostfeld and Keesing [24] proposed that pathogens transmitted by highly non-specific vectors might adapt to the hosts they are most likely to experience. This process would be akin to adaptation by passively-dispersed, free-living organisms to the habitat type they are most likely to encounter. They hypothesized that hosts that are widespread and abundant might be encountered by multi-host pathogens more frequently, leading to pathogen adaptation to exploit most common hosts. Such adaptation would produce high reservoir competence. Clearly though, the potential exists for both host life history traits and pathogen adaptation to affect variation in reservoir competence among host species. In this study we used a model selection approach to ask whether reservoir competence for three emerging tick-borne pathogens is more strongly correlated to: (1) life-history traits (i.e. fast pace of life predicts high competency), or (2) ecological traits (i.e., probability of encounter with infected ticks predicts high competency).

## Materials and Methods

### Life history traits

This analysis was conducted to ask whether reservoir competence for the set of tick-borne, zoonotic pathogens is predictable based on life-history features of the host, under the assumption that life history features influence defense against these pathogens (see Introduction). We began with the set of life history traits selected by Huang et al. [22], namely, adult body mass, gestation length, and litter size, for each of the nine mammalian hosts for which we have gathered data on reservoir competence (see below). We note that body size is, strictly speaking, not a life history trait, although it impacts many life history variables that influence the pace of life [25]. Given little knowledge on which to base hypotheses linking specific life history traits to specific immune traits, we take an inclusive approach here. Following Huang et al. [22], we log transformed gestation length and body mass. We conducted a second, more comprehensive analyses of pace of life impacts on competence that included adult body mass, gestation length, offspring produced per year, age at maturity, longevity, and log (basal metabolic rate * g^−1^) (Table S1). Life history data were taken from the PanTHERIA database of mammal life history [26]. For eastern chipmunks and white-tailed deer, data on basal metabolic rate were not available in PanTHERIA, so we obtained relevant data from AnAge, the animal aging and longevity database (http://genomics.senescence.info/species/). Because many life history traits are correlated, we used principal components analysis to derive a set of new orthogonal variables that maximized the variance explained. We then conducted linear regressions with the first and second principal components as independent variables and realized reservoir competence as the dependent variable. We performed these analyses for each of the three tick-borne pathogens separately.

Because we were using host species as our units of comparison, the potential exists for inflating degrees of freedom if particular species’ traits are not independent of one another by virtue of phylogenetic similarity [27]. For our group of hosts we think this potential is low, because the maximum number of species per order is three (Rodentia), per family is two (two each for Soricidae and Sciuridae), and per genus is one. Similar analyses of life history correlates of reservoir competence for Lyme disease by Huang et al. [22] showed only modest quantitative (not qualitative) effects of using a phylogenetic correction, and that the uncorrected (conventional) analyses were conservative. For these reasons and for transparency of interpretation, we have chosen to present the uncorrected results. Nevertheless, in Supplementary Online materials, we present results of analyses using a phylogenetic correction based on a cladogram (Fig. S1) for the nine species we analyzed (Table S3).

### Surrogates for tick/pathogen encounter rates

These variables were included to address whether reservoir competence for the tick-borne pathogens is potentially affected by the ability of the pathogens to adapt to the hosts they are most likely to encounter. We have no direct measurement of encounter rates between ticks or tick-borne pathogens and hosts, so we chose two surrogates. First, we assumed that overall encounter rates between ticks and a host species are a function of the average population density of that host. We base this assumption on the observation that blacklegged ticks are “sit-and-wait” parasites that move very little on their own and depend on host movements in order to gain access to a blood meal [28]. We are aware that host species-specific features other than population density, such as movement patterns, might additionally affect encounter rates with ticks, but we were unable to determine which behaviors might be important or to find relevant data for this community of hosts.

Although host population density might govern that species’ overall encounter rates with ticks, prior research shows that a large proportion of ticks encountering a host fail to feed to repletion, with some hosts grooming off and killing many ticks [29]. The product of tick encounter rates and the proportion of those ticks that survive to repletion (i.e., permissiveness of that host species; [29] determines the natural “body burden”, defined as the average number of ticks that feeds from a member of each host species under natural conditions [29]. Therefore, the average body burden on a host species times the average population density of that host determines the total number of ticks that feeds on that population of hosts in nature. Consequently, for our second surrogate for tick-host encounter rates we used host density * body burden, which probably is correlated with the opportunity for a tick-borne pathogen to invade a host species. Ideally, body burden data would be specific to the nymphal stage, because this is the stage responsible for most transmission events. However, in the absence of data on nymphal body burdens, we relied on our field data on larval body burdens for the nine mammal species [5], [29]. Values and data sources for the demographic variables density and density * burden are given in Table S2.

### Comparing models with life history traits and tick/pathogen encounter rates

We then assessed levels of support for models in which reservoir competence was a function of specific life history traits or our surrogates for tick-pathogen encounter rates, or both. We used model comparison procedures to ask which combinations of the life history variables (the more comprehensive set), principal component scores, average population densities, and density * body burden were best supported by the data for each of the three pathogens separately. We used AIC corrected for small sample sizes (AIC_c_) and Akaike weights to compare levels of support for each model, including those models with at least 10% weight.

### Reservoir competence

Extensive studies have been undertaken to determine realized reservoir competence for a large component of the host community in an endemic zone for Lyme disease in southeastern New York State. Although these studies are specific to one site, the location is representative of the northeastern region of the United States with high Lyme disease incidence. Methods have been described in detail in [3]–[5]. We use previously published data for *B. burgdorferi* [5], *B. microti* [3], and the strain of *A. phagocytophilum* that infects humans (ha strain: [6]). Reservoir competence values for the three tick-borne pathogens are given in Table S2.

## Results

### Life history correlates

**Figure 1:**
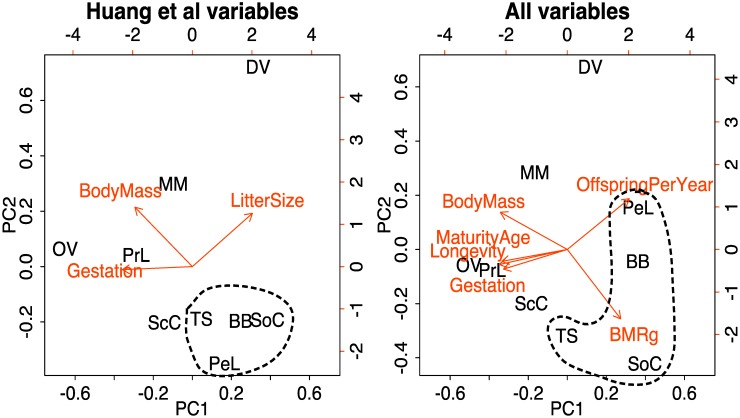
Results of principle components analysis on life history variables used by Huang et al. [22] and in the more inclusive data set (all variables). Vectors show the mapping of the original variables onto the first two principle component axes (PC1 and PC2). Host species are given by the following notation: OV = *Odocoileus virginianus*; PrL = *Procyon lotor*; MM = *Mephitis mephitis*; DV = *Didelphis virginiana*; ScC = *Sciurus carolinensis*; TS = *Tamias striatus*; BB = *Blarina brevicauda*; SoC = *Sorex cinereus*; and PeL = *Peromyscus leucopus*.

Principle components 1 and 2, respectively, explained 76.8% and 20.6% of the variation for the life history data set consisting of the three variables used by Huang et al. [22], and 74.5% and 15.3% of the variation for the more complete life-history data set (Fig. 1). For the 3-variable data set, high values of PC1 represented small bodies, short gestation, and large litters, whereas low values for PC2 represented small bodies and small litters. Mice, chipmunks, and the two shrew species were clustered in the lower right quadrant of Fig. 1A, representing fast life histories. For the larger data set, high values of PC1 were similar to the smaller data set (small bodies, short gestation, and many offspring per year) but additionally represented young age at maturity, short life span, and high mass-specific basal metabolic rate (BMR). Low values of PC2 were best represented by low BMR and few offspring/year (Fig. 1B). The four smallest mammals were clustered at high values of PC1 but spread more widely across the PC2 axis.

**Figure 2:**
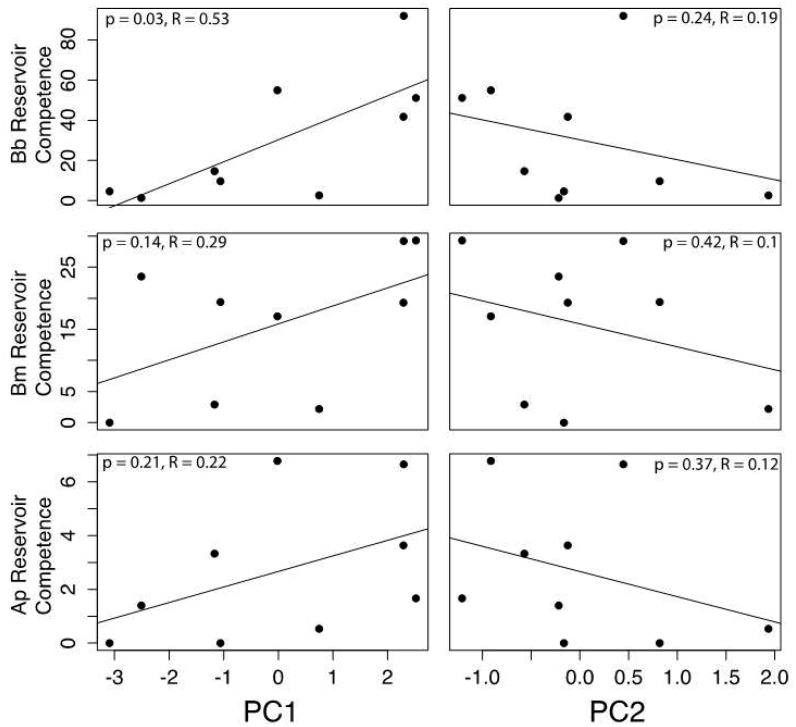
Relationship between a host species’ score on the ordination of six life history traits, represented by two principal components (PC1 and PC2) and that host’s reservoir competence for *Borrelia burgdorferi* (Bb), *Babesia microti* (Bm), and the human-infectious strain of *Anaplasma phagocytophilum*.

For the 3-variable life history data set, multiple regression revealed that reservoir competence for *B. burgdorferi* was positively correlated with PC1 (t = 2.4, P = 0.05) and negatively correlated with PC2 (t = −3.3, P = 0.01). Reservoir competence for *A. phagocytophilum* was negatively correlated with PC2 (t = −2.6, P = 0.04) and uncorrelated with PC1 (t = 1.2, P = 0.27). For *B. microti* reservoir competence was not predicted by species scores on either of the principal components axes (PC1: t = 1.4, P = 0.206; PC2: t = −1.599, P = 0.16) (Fig. 2). For the 6-variable life history data set, multiple regression showed that reservoir competence for *B. burgdorferi* was positively correlated with PC1 (t = 3.0, P = 0.02), but uncorrelated with PC2 (t = −1.2, P = 0.27) (Table 1). *B. microti* and *A. phagocytophilum* were not correlated with either PC1 (*B. microti*: t = 1.7, P = 0.15; *A. phagocytophilum*: t = 1.4, P = 0.21) or PC2 (*B. microti*: t = −1.0, P = 0.37; *A. phagocytophilum*: t = −1.0, P = 0.35).

**Table 1:** Multiple regression results for analyses of the relationship between the two principal components (PC1 and PC2) describing host life history and reservoir competence for three tick-borne pathogens.

	Huang et al. variables					All variables				
	PC1		PC2			PC1		PC2		
Pathogen	b	t	b	t	R^2^	b	t	b	t	R^2^
*B. burgdorferi*	10.4	2.4*	−27.8	−3.3*	0.74	11.0	3.0*	−9.9	−1.2	0.63
*B. microti*	3.3	1.4	−7.2	−1.6	0.43	2.9	1.7	−3.7	−1.0	0.38
*A. phagocytophilum*	0.6	1.2	−2.3	−2.6*	0.58	0.6	1.4	−0.9	−1.0	0.33

The correlation coefficients for each independent variable (b), corresponding t-value (*P≤0.05), and whole model R-square values are given. “Huang et al. variables” refers to the life history traits used by Huang et al. [22], whereas “All variables” refers to the larger data set.

### Demographic correlates

**Figure 3:**
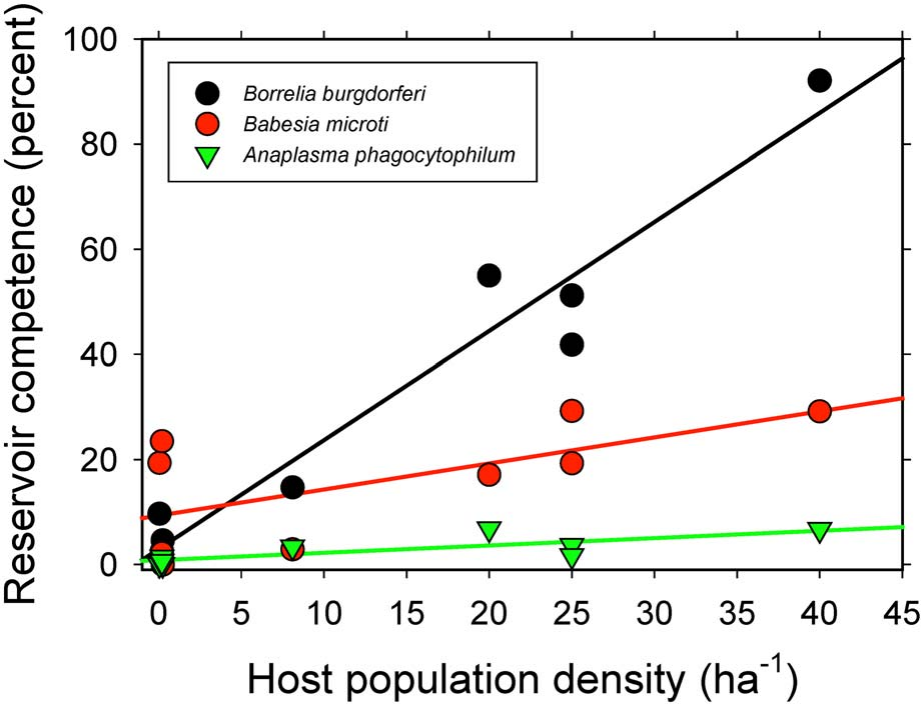
Relationship between average host population density [5] and reservoir competence of that host species. Host population density is used as one of two proxies for encounter rates between ticks and host species.

For *B. burgdorferi* and *A. phagocytophilum* we observed a strong positive correlation between population density of a host and its reservoir competence (t = 11.4, P<0.001; and t = 3.4, P = 0.01, respectively). A similar, positive relationship was also apparent for *B. microti*, but the correlation was marginally non-significant (t = 2.2, P = 0.06) (Fig. 3).

**Figure 4:**
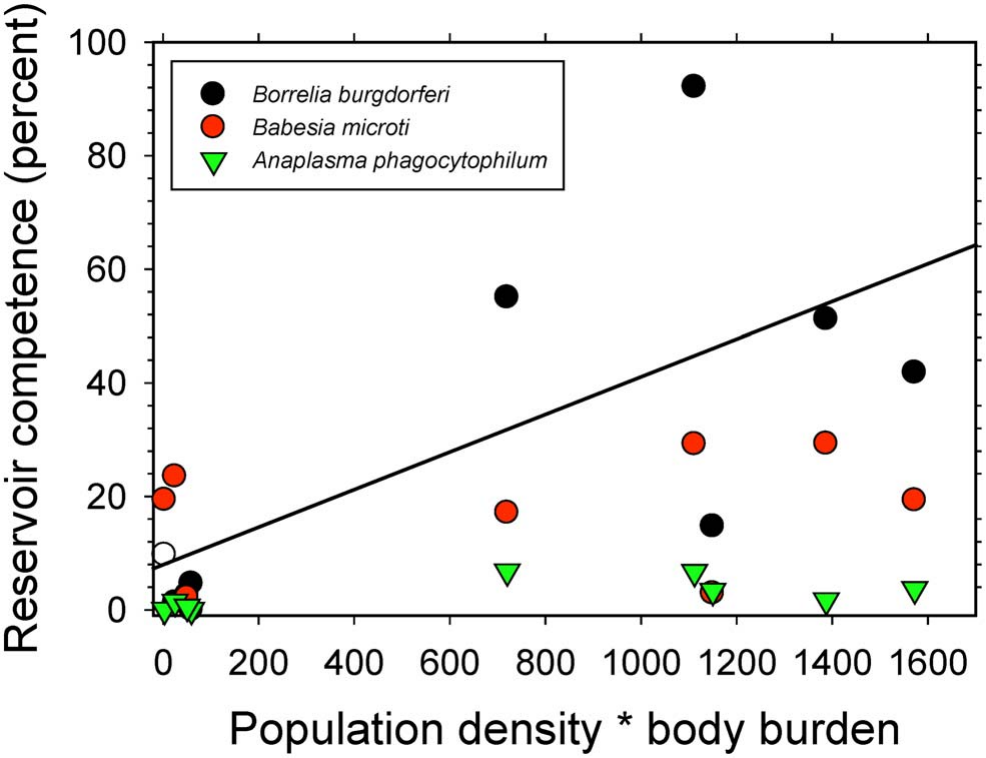
Relationship between the product of average host population density and measured body burden (number of naturally occurring larval ticks per host individual) and reservoir competence of that host species. The product of population density and body burden, which represents the average number of ticks fed by a particular population of hosts, is used as one of two proxies for encounter rates between ticks and host species.

We found that reservoir competence for *B. burgdorferi* was significantly correlated with host density * tick burden (t = 2.5, P = 0.04), but reservoir competence for *B. microti* and *A. phagocytophilum* were not, although we observed a marginally non-significant positive correlation in the case of *A. phagocytophilum* (t = 1.9, P = 0.10) (Fig. 4).

### Model comparisons

For each of the three pathogens, we computed AIC_c_ values and Akaike weights for multiple regressions that included all combinations of the first two principal component axes (PC1 and PC2), the six life history predictors, and both demographic predictor variables. For *B. burgdorferi*, the best-supported models were those including population density (positive correlation) plus density * body burden (positive) (52.3% model weight) and density alone (33.3% model weight) (Table 2). For *B. microti*, the best-supported models were those including body mass (negative), the null (intercept only) model, and density (positive), with 20.2%, 14.5%, and 14.3% weight, respectively. For *A. phagocytophilum*, the best supported model was that including density (positive: 44.8% model weight) followed by density plus age at maturity (positive; 8.6% model weight). No other models had more than 10% model weight.

**Table 2:** Results of model comparisons in which all the two principal component axes, PC1 and PC2, as well as the individual life history traits and the two surrogates of pathogen-host encounter probabilities are incorporated as explanatory variables for reservoir competence.

Pathogen	Best models (coefficient)	AICc	ModelWeight (%)	Model R^2^
*Borrelia burgdorferi*	Density (2.58)Burden*Density (−0.014)	69.76	52.3	0.98
	Density (2.075)	70.66	33.3	0.95
*Babesia microti*	Body Mass (−2.39)	73.71	20.2	0.27
	Intercept	74.37	14.5	n/a
	Density (0.50)	74.40	14.3	0.41
*Anaplasma phagocytophilum*	Density (0.14)	44.07	44.8	0.62
	Density (0.22) Age atMaturity (0.01)	47.38	8.6	0.75

The coefficients for each explanatory variable, AICc values (corrected for small samples), and model weights are provided for at least the top two models and/or all models with ≥10% AIC weight.

## Discussion

For a suite of three tick-borne zoonotic pathogens in eastern North America, two small rodents and two small soricimorphs consistently are among the most competent natural reservoirs for tick infection. Although many other species of terrestrial vertebrates are commonly exposed to these pathogens, they are comparatively inefficient reservoirs, infecting only a small proportion of the ticks that feed on them [11], [29]. Following the analysis of Lyme disease reservoirs by Huang et al. [22], we asked whether life history features of the members of this community of mammalian hosts for blacklegged ticks predict their reservoir competence for two other quite distinct pathogens. We also asked whether two measures of host-tick encounter probability influence reservoir competence. For all three pathogens we found that high average population density and “fast” life history features were predictors of high reservoir competence. However, the importance of life history traits and the specific traits linked to high reservoir competence varied among the pathogens. In the case of *B. burgdorferi*, model comparison revealed that all of the best-supported models (>10% Akaike weights) included high population density as a determinant of high reservoir competence, with some support for models including high basal metabolic rate. In contrast, for *B. microti*, small body mass, and to a lesser extent high population density were supported as determinants of reservoir competence. For this pathogen, the null model was almost equally as well supported as were models with body mass and population density. High population density was again supported for models of *A. phagocytophilum*, with early age at maturity also correlated with high reservoir competence. Surprisingly, neither of the composite life-history variables, represented as principal component axes, entered any of the best-supported models for any of the three pathogens.

### Host life history and reservoir competence

Our results based on life history traits alone suggested that high reservoir competence for *B. burgdorferi*, and possibly *A. phagocytophilum*, was associated with high BMR and many offspring/year, characteristic of species with fast life histories. This conclusion is broadly consistent with research by Huang et al. [22], who compared reservoir competence metrics across mammalian and avian communities and found that hosts with faster life histories were better reservoirs for the agent of Lyme disease, West Nile virus, and Eastern Equine Encephalitis virus. These results are also consistent with similar analyses of an aphid-transmitted multi-host pathogen (barley-cereal yellow dwarf virus) of herbaceous plants [17] and trematode parasites of amphibians [20], showing that fast-lived host species were more prone to infection or high reservoir potential.

Fast life history strategies are thought to represent heavy investment in rapid growth, maturity, and reproduction at the expense of other factors, namely immune defenses [12], [13], [16], [30]–[32]. According to the “ecoimmunological pace of life” hypothesis [12], [33], [34], as a result of trade-offs between pace of life and self-defense, fast-lived species should be less resistant to infection and proliferation by some pathogens, which could increase reservoir competence [16], [32], [35], [36]. Alternatively, fast-lived species might tend to be more tolerant of infections [37], [38], but see [20]. Regardless, identifying life history features that predict reservoir competence for multi-host pathogens will help in predicting the consequences of changing host communities for disease risk [18], [21], [22]. Ideally, the search for predictive life history features would be informed by more thorough knowledge of specific relationships and trade-offs between immune functions and determinants of survival rates and fecundity.

Future research mechanistically linking life history features to reservoir competence for these pathogens should address two key questions: (1) which specific life history traits influence immune functions; and (2) which specific immune traits determine reservoir competence? Because experimentally manipulating multiple life history features, independently and in combination, seems infeasible, comparative approaches based on large samples of hosts are warranted. Similarly, challenges in manipulating multiple immune traits within hosts would appear to dictate a comparative approach using many species or populations of hosts.

### Host availability and reservoir competence

We found support for a positive relationship between host population density and reservoir competence for all three pathogens. This result is consistent with the hypothesis that multi-host pathogens evolve to exploit the hosts that they are most likely to encounter [20], [24], [39]. We found only weak support for the hypothesis that reservoir competence correlates with total tick feeding opportunities, represented by the product of host density and tick burdens. It is important to note that population density and body mass are inversely correlated for our group of hosts. In fact, it is often the case that species with fast life histories tend to occur at higher population densities than their slower-lived counterparts [25]. Therefore, it is not possible to distinguish between features intrinsic to hosts (e.g., life history and immune strategies) and extrinsic features that represent pathogen-encounter probabilities (e.g., host density and tick burden). It is entirely plausible that high reservoir competence is caused both by pathogen adaptation to frequently-encountered hosts and fast-paced hosts being more poorly defended against pathogens. In addition, collinearity among independent variables complicates our ability to interpret results. Although we used ordination to replace collinear life history variables with orthogonal ones, none of the principal components explained patterns of reservoir competence as well as some of the original variables. Quantifying the relative importance of these potential drivers of the association between small-bodied, abundant species and reservoir competence will require a concerted effort to determine how pathogen adaptation and host immune functions independently and together determine reservoir competence.

In addition to limited ability to infer causality from correlations, a key caveat of our approach is the use of average values among individuals to estimate reservoir competence, life history traits and demographic features. Heterogeneities among individuals (and populations) certainly exist and should be incorporated in future efforts [32]. For example, intraspecific variation in reservoir competence for *B. burgdorferi* has been described [11], [40], but individual heterogeneities in life history from these same populations have not. Variance in host traits by itself could be an important determinant of reservoir competence at the population level. For instance, a host species with high mean susceptibility but low variance might strongly foster infection and proliferation by a pathogen, whereas a species with similar mean but high variance might provide fewer opportunities for pathogen transmission [41], [42]. Simultaneous estimates of reservoir competence, life history, and demography pertaining to individual hosts and host populations would facilitate stronger inference.

The patterns linking demographic and life history features with reservoir competence will be less likely to apply to specialist pathogens than to the generalist pathogens we analyzed. Strict specialists will experience only one host; therefore, interspecific comparisons among host traits will not be relevant to transmission dynamics. On the other hand, such comparisons might pertain if specialist pathogens frequently evolve from more generalized ancestors [43]. With ample opportunity to infect multiple species of host, a generalist pathogen will be likely to experience trade-offs that affect its ability to colonize and proliferate within each host [23]. Such trade-offs could contribute to increasing specialization that would then reduce selection pressures from other (non-specialized) hosts.

One potential application of our results is increased ability to predict which hosts might act as “keystone reservoirs” when generalist pathogens emerge. Our results suggest that of the host species that might potentially be exposed, those with comparatively high population densities, small bodies, and fast pace of life will often be keystone reservoirs that should be targeted for surveillance or management.

## Conclusions

We examined the traits of mammals that correlate with competence in transmitting zoonotic, tick-borne pathogens to feeding tick vectors. We found support for the hypothesis that the most competent wildlife reservoirs for three common tick-borne pathogens are hosts that tend to occur at high population densities and have fast life histories compared to other mammalian hosts that are poorer reservoirs. However, the specific traits that correlated best with reservoir competence for the agents of Lyme disease, babesiosis, and anaplasmosis differed to some degree. The relative importance of extrinsic (population density) vs intrinsic (life history) features remains to be fully determined, although we found stronger support for the former. Specific causal relationships between demography, life history, immune functions, and reservoir competence, are important frontiers for future research.

## Supporting Information

Figure S1 **Phylogeny of the mammalian hosts for tick-borne zoonotic pathogens analyzed for their life history features, tick-encounter surrogates, and reservoir competence.** A phylogenetic correction, based on this cladogram, was applied before conducting the analyses the results of which are provided in Table S3.
(TIF)

Table S1 **Data from PanTHERIA and AnAge used in the analyses of effects of life history variables on reservoir competence for the three tick-borne pathogens.**
(DOCX)

Table S2 **Data for each of the nine hosts analyzed for impacts of life history variables and tick-encounter surrogates.** RC = reservoir competence for *Borrelia burgdorferi, Babesia microti,* and the human-infectious (ha) strain of *Anaplasma phagocytophilum*.
(DOCX)

Table S3 **Results of model comparisons in which all the two principal component axes, PC1 and PC2, as well as the individual life history traits and the two surrogates of pathogen-host encounter probabilities are incorporated as explanatory variables for reservoir competence.** This analysis differs from that summarized in the text and Table 2 in that a phylogenetic correction (see Materials and Methods) was conducted prior to analysis. The coefficients for each explanatory variable, AICc values (corrected for small samples), and model weights are provided for at least the top two models and all models with ≥10% AIC weight.
(DOCX)
