# Supplementary figures and images for: Life History and Demographic Drivers of Reservoir Competence for Three Tick-Borne Zoonotic Pathogens
Source: PLoS One. 2014 Sep 18;9(9):e107387. doi: 10.1371/journal.pone.0107387 (PMC4169396; doi:10.1371/journal.pone.0107387)

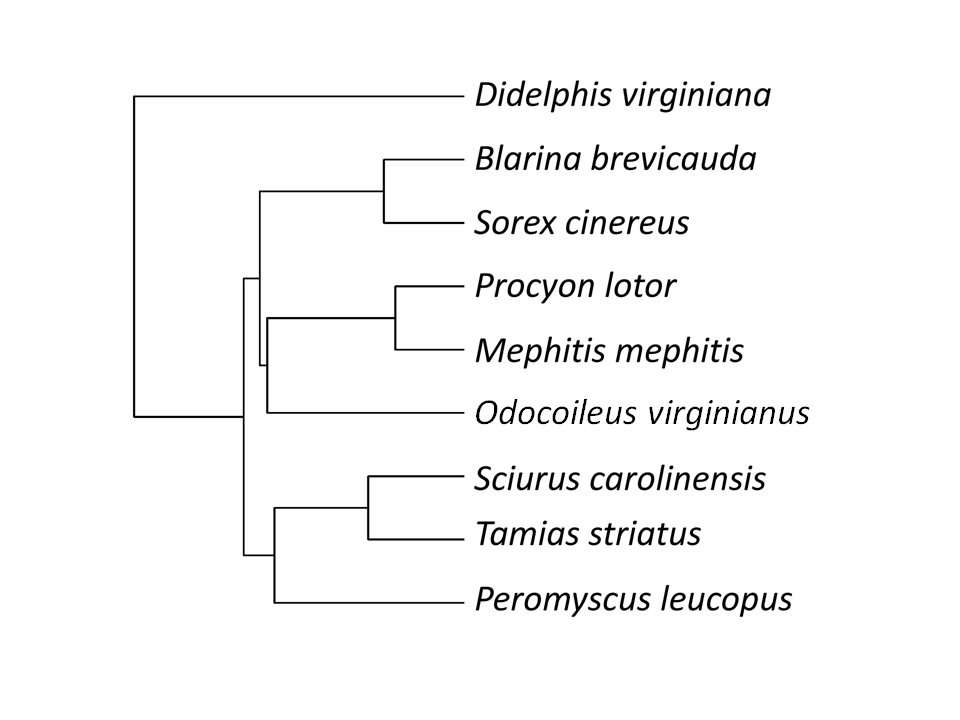

Supplement: Figure S1 — Phylogeny of the mammalian hosts for tick-borne zoonotic pathogens analyzed for their life history features, tick-encounter surrogates, and reservoir competence. A phylogenetic correction, based on this cladogram, was applied before conducting the analyses the results of which are provided in Table S3. (TIF) [file pone.0107387.s001.tif]
