# Supplementary material for: Life History and Demographic Drivers of Reservoir Competence for Three Tick-Borne Zoonotic Pathogens
Source: PLoS One. 2014 Sep 18;9(9):e107387. doi: 10.1371/journal.pone.0107387 (PMC4169396; doi:10.1371/journal.pone.0107387)
Supplement: Table S1 — Data from PanTHERIA and AnAge used in the analyses of effects of life history variables on reservoir competence for the three tick-borne pathogens. (DOCX) [file pone.0107387.s002.docx]

**Table S1.** Data from PanTHERIA and AnAge used in the analyses of effects of life history variables on reservoir competence for the three tick-borne pathogens.

| **Species** | **Log Body**  **Mass (g)** | **Log Gestation**  **Period (d)** | **Maturity**  **Age (d)** | **Max Longevity (mo)** | **Offspring per year** | **Log**  **BMR/g** |
| --- | --- | --- | --- | --- | --- | --- |
| *Mephitis mephitis* | 7.78 | 4.15 | 355.18 | 155 | 11.40 | -1.32 |
| *Procyon lotor* | 8.76 | 4.17 | 561.28 | 252 | 3.06 | -0.96 |
| *Peromyscus leucopus* | 2.89 | 3.14 | 45.15 | 38 | 19.22 | 0.46 |
| *Blarina brevicauda* | 2.92 | 3.02 | 71.19 | 33 | 16.17 | 1.07 |
| *Sorex cinereus* | 1.44 | 2.90 | 147.56 | 23 | 12.98 | 2.19 |
| *Tamias striatus* | 4.70 | 3.43 | 365.00 | 96 | 4.20 | 0.49 |
| *Odocoileus virginianus* | 11.24 | 5.31 | 365.00 | 276 | 1.57 | -1.02 |
| *Didelphis virginiana* | 7.80 | 2.54 | 225.55 | 60 | 17.240 | -1.41 |
| *Sciurius carolinensis* | 6.30 | 3.80 | 337.88 | 288 | 5.960 | -0.12 |
