# Supplementary material for: Life History and Demographic Drivers of Reservoir Competence for Three Tick-Borne Zoonotic Pathogens
Source: PLoS One. 2014 Sep 18;9(9):e107387. doi: 10.1371/journal.pone.0107387 (PMC4169396; doi:10.1371/journal.pone.0107387)
Supplement: Table S2 — Data for each of the nine hosts analyzed for impacts of life history variables and tick-encounter surrogates. RC = reservoir competence for Borrelia burgdorferi, Babesia microti, and the human-infectious (ha) strain of Anaplasma phagocytophilum. (DOCX) [file pone.0107387.s003.docx]

**Table S2.**  Data for each of the nine hosts analyzed for impacts of life history variables and tick-encounter surrogates. RC = reservoir competence for *Borrelia burgdorferi, Babesia microti,* and the human-infectious (ha) strain of *Anaplasma phagocytophilum*.

| **Species** | **Density (indv/ha)** | **Body Burden (ticks/host)** | **RC**  ***B. burgdorferi*** | **RC**  ***B. microti*** | RCRC **RC**  **RC *A. phagocytophilum***  **A.** |
| --- | --- | --- | --- | --- | --- |
| *Mephitis mephitis* | 0.05 | 66.8 | 9.7 | 19.4 | 0 |
| *Procyon lotor* | 0.20 | 127 | 1.3 | 23.5 | 1.4 |
| *Peromyscus leucopus* | 40 | 27.8 | 92.1 | 29.2 | 6.7 |
| *Blarina brevicauda* | 25 | 62.9 | 41.8 | 19.3 | 3.6 |
| *Sorex cinereus* | 25 | 55.5 | 51.2 | 29.3 | 1.7 |
| *Tamias striatus* | 20 | 36 | 55 | 17.1 | 6.8 |
| *Odocoileus virginianus* | 0.25 | 239 | 4.6 | 0 | 0 |
| *Didelphis virginiana* | 0.20 | 254 | 2.6 | 2.2 | 0.5 |
| *Sciurius carolinensis* | 8.10 | 142 | 14.7 | 2.9 | 3.3 |
