# Supplementary material for: Life History and Demographic Drivers of Reservoir Competence for Three Tick-Borne Zoonotic Pathogens
Source: PLoS One. 2014 Sep 18;9(9):e107387. doi: 10.1371/journal.pone.0107387 (PMC4169396; doi:10.1371/journal.pone.0107387)
Supplement: Table S3 — Results of model comparisons in which all the two principal component axes, PC1 and PC2, as well as the individual life history traits and the two surrogates of pathogen-host encounter probabilities are incorporated as explanatory variables for reservoir competence. This analysis differs from that summarized in the text and Table 2 in that a phylogenetic correction (see Materials and Methods) was conducted prior to analysis. The coefficients for each explanatory variable, AICc values (corrected for small samples), and model weights are provided for at least the top two models and all models with ≥10% AIC weight. (DOCX) [file pone.0107387.s004.docx]

**Table S3.** Results of model comparisons in which all the two principal component axes, PC1 and PC2, as well as the individual life history traits and the two surrogates of pathogen-host encounter probabilities are incorporated as explanatory variables for reservoir competence. This analysis differs from that summarized in the text and Table 2 in that a phylogenetic correction (see Materials and Methods) was conducted prior to analysis. The coefficients for each explanatory variable, AICc values (corrected for small samples), and model weights are provided for at least the top two models and all models with ≥10% AIC weight.

| **Pathogen** | **Best models (coefficient)** | **AICc** | **Model Weight (%)** |
| --- | --- | --- | --- |
| *Borrelia burgdorferi* | Density (2.58)  Burden*Density (-0.014) | 31.06 | 56.1 |
|  | Density (2.075) | 31.99 | 35.3 |
| *Babesia microti* | Body Mass (-2.09) | 24.33 | 43.3 |
|  | PC1 (-0.35) | 27.20 | 10.3 |
|  | Density (0.39) | 27.53 | 8.7 |
| *Anaplasma phagocytophilum* | Intercept | 9.89 | 40.7 |
|  | Density (0.11) | 11.23 | 20.9 |
